# Prevalence of Human Immunodeficiency Virus, Hepatitis B and Hepatitis C Virus Antibodies and Hepatitis B Antigen Among Commercial Sex Workers in Japan

**DOI:** 10.1155/S1064744901000357

**Published:** 2001

**Authors:** Kazuhisa Ishi, Fujihiko Suzuku, Akira Saito, Shinsaku Yoshimoto, Takeyoshi Kubota

**Affiliations:** 1 Department of Clinical Pathology Juntendo University Urayasu Hospital Chiba Japan; 2 Department of Obstetrics and Gynecology Juntendo University Urayasu Hospital 2-1-1 Tomioka Urayasu City Chiba Japan

## Abstract

*Objective:* To investigate the prevalence of antibodies to human immunodeficiency virus (HIV), hepatitis B virus (HBV) and hepatitis C virus (HCV), and of hepatitis B surface (HBs) antigen in commercial sex workers (CSW) who attended a sexually transmitted disease (STD) clinic in Tokyo.

*Methods:* Surveys were conducted on 308 CSW and 384 control subjects for HIV antibody or 241 control subjects for HBs antibody and antigen and HCV antibody.

*Results:* HIV antibodywas not detected in either CSW or control subjects. The positive rates for HBs antigen and antibody were 0.6 and 23.4%, respectively, in the CSW group, and 0.4 and 71.8% in the control group. The HCV antibody positive rate was 3.2% in the CSW group and 0.4% in the control group.

*Conclusion:* A statistically significant difference between the two groups was observed only in HCV antibody positive rate. STD checkup for CSW alone is inadequate – STD health education and screening for the general public are also required.


*Infect Dis Obstet Gynecol 2001;9:215–219*

### Prevalence of human immunodeficiency virus, hepatitis B and hepatitis C virus antibodies and hepatitis B antigen among commercial sex workers in Japan

**Kazuhisa Ishi[1], Fujihiko Suzuku[1], Akira Saito[1], Shinsaku Yoshimoto[1] and Takeyoshi Kubota[2]**

[1]*Department of Clinical Pathology, Juntendo University Urayasu Hospital, Chiba, Japan*
[2]*Department of Obstetrics and Gynecology, Juntendo University Urayasu Hospital, Chiba, Japan*

***Objective:*** To investigate the prevalence of antibodies to human immunodeficiency virus (HIV), hepatitis B virus (HBV) and hepatitis C virus (HCV), and of hepatitis B surface (HBs) antigen in commercial sex workers (CSW) who attended a sexually transmitted disease (STD) clinic in Tokyo.
***Methods:*** Surveys were conducted on 308 CSW and 384 control subjects for HIV antibody or 241 control subjects for HBs antibody and antigen and HCV antibody.
***Results:*** HIV antibody was not detected in either CSW or control subjects. The positive rates for HBs antigen and antibody were 0.6 and 23.4%, respectively, in the CSW group, and 0.4 and 71.8% in the control group. The HCV antibody positive rate was 3.2% in the CSW group and 0.4% in the control group.
***Conclusion:*** A statistically significant difference between the two groups was observed only in HCV antibody positive rate. STD checkup for CSW alone is inadequate – STD health education and screening for the general public are also required.

Key words: HUMAN IMMUNODEFICIENCY VIRUS (HIV); HEPATITIS B VIRUS (HBV); HEPATITIS C VIRUS (HCV); COMMERCIAL SEX WORKERS (CSW)

### INTRODUCTION

The term sexually transmitted disease (STD) includes all infections transmitted by sexual contact, and thus describes a variety of diseases. In Japanese men and women, nongonococcal urethritis or cervicitis is the most common STD, caused mainly by *Chlamydia trachomatis*, followed by *Neisseria gonorrhoeae* infection in men and herpes simplex in women[1,2]. In addition, asymptomatic infections caused by a variety of pathogens – including *C. trachomatis* and human papilloma virus (HPV) – are common in Japan, and infection with human immunodeficiency virus (HIV) is also on the increase. Hepatitis B virus (HBV) and hepatitis C virus (HCV) infections are often asymptomatic in the early stages, and transmission of these viruses unconsciously to sexual partners by sexual contact is not uncommon. HBV has been reported to be transmitted among husbands and wives and lovers, resulting in fulminating hepatitis. Furthermore, high prevalence of HBV antibodies and antigen and HCV antibody has been reported among commercial sex workers (CSW) and homosexuals[2].

Correspondence to: Kazuhisa Ishi, Department of Clinical Pathology, Juntendo University Urayasu Hospital, 2-1-1 Tomioka, Urayasu City, Chiba, Japan Email: ishi3988@kt.rim.or.jp

In the present study, we examined the prevalence of antibodies to HIV, HBV and HCV and Hepatitis B surface (HBs) antigen in CSW, although these infection rates are expected to be lower than those of *C. trachomatis*, *N. gonorrhoeae* and HPV.

### MATERIALS AND METHODS

Three hundred and eight women (applicants for regular STD screening, age range 19–41 years, mean age 28.2 years) who attended an STD clinic in an entertainment area in Tokyo were studied. The subjects were all Japanese CSW who had worked for a duration of between 6 months and 15 years. Not all of them worked constantly in the same entertainment area. Some had moved recently from another area in Japan. Two control groups were used: the control group for HIV antibody consisted of 384 pregnant women (age range 21–38 years, mean age 30.5 years) who attended the Department of Obstetrics at Juntendo University Urayasu Hospital, and the control group for HBs antibody and antigen and HCV antibody consisted of 241 female staff (age range 20–45 years, mean 29.5 years) of Juntendo University Medical School Hospital (Hongo, Tokyo) and Juntendo University Urayasu Hospital who had no contact with patients, thus excluding medical doctors, nurses and medical technologists.

HIV, HBV and HCV screening tests were performed by the chemiluminescent enzyme immunoassay (EIA) (Lumipulse *f*, Fujirebio Inc., Japan) using HIV recombinant antigen (HIV-1/2, Ortho Inc., Japan), HCV recombinant antigen (third generation, Ortho Inc., Japan), and HBs antigen and antibody (Fujirebio Inc., Japan). The Lumipulse *f* system is a commercially-available chemiluminescent EIA method[3] that is the second most widely used method in Japan.

The data were analyzed statistically using Fisher's direct test. A $p$ value of less than 0.05 was considered significant.

### RESULTS

HIV antibody was not detected in either STD clinic attendants (CSW group) or control subjects.

The positive rates for HBs antigen and antibody were 0.6 and 23.4% respectively in the CSW group, and 0.4 and 71.8% in the control group. The HCV antibody positive rate was 3.2% in the CSW group and 0.4% in the control group. A statistically significant difference between the two groups was observed only in HCV antibody positive rate ($p < 0.01$) (Table 1).

Liver function and other STD complications were examined in subjects positive for HCV antibody or HBs antigen. Among the ten HCV antibody-positive CSW, four had impaired liver function – defined as > 32 IU/l aspartate aminotransferase (AST) and/or > 42 IU/l alanine aminotransferase (ALT) – and three were complicated by other STD (one case positive for syphilis, one case positive for *C. trachomatis* and *N. gonorrhoeae*, and one case positive for HBs antigen). The two CSW positive for HBs antigen had no abnormal liver function and one was positive for *C. trachomatis*. Information regarding liver function was not available for the one control subject positive for HBs antigen (Table 2). None of the CSW reported a history of blood transfusion or drug abuse when asked during history taking.

**Table 1** Prevalence of HIV, HBs and HCV antibody and HBs antigen

| | *Antibody* | | | | | | *Antigen* | | |
|---|---|---|---|---|---|---|---|---|---|
| | *HIV cases* | | *HBs cases* | | *HCV cases* | | *HBs cases* | | *Sample size* |
| CSW | 0 | (0%) | 72 | (23.4%) | 10 | (3.2%) | 2 | (0.6%) | 308 |
| Controls | 0 | (0%) | 173 | (71.8%) | 1 | (0.4%) | 1 | (0.4%) | 384/241* |
| Total | 0 | (0%) | 245 | (44.6%) | 11 | (2.0%) | 3 | (0.5%) | 692/549* |
| | | | | | $p < 0.01$ | | | | |

HIV, human immunodeficiency virus; HBs, hepatitis B surface antigen; HCV, hepatitis C virus; CSW, commercial sex workers; *total 384 HIV controls, 241 HBs/HCV controls

**Table 2** Liver function and other STD complications in subjects positive for HCV antibody or HBs antigen

| | | | | Liver function | | | | | |
|---|---|---|---|---|---|---|---|---|---|
| | | *Case No.* | *Age (y)* | *AST* (IU/l) | *ALT* (IU/l) | *T-bil* (mg/dl) | *Work duration (y)* | *Other STD* | *Condom use* |
| HCVAb | CSW | 1 | 34 | 27 | 38 | 1 | 10 | Syphilis | No |
| | | 2 | 28 | 143 | 191 | 0.6 | 0.5 | | No |
| | | 3 | 29 | 40 | 65 | 0.3 | 1 | | No |
| | | 4 | 32 | 26 | 31 | 0.5 | 1.5 | | No |
| | | 5 | 29 | 20 | 13 | 0.8 | 3 | | No |
| | | 6 | 30 | 17 | 21 | 0.3 | 3 | Ct Ng | No |
| | | 7 | 29 | 47 | 55 | 0.2 | 2 | | No |
| | | 8 | 40 | 24 | 22 | 0.3 | 9 | HBsAg+ | No |
| | | 9 | 28 | 55 | 70 | 0.7 | 6 | | No |
| | | 10 | 38 | 32 | 22 | 0.2 | 15 | | No |
| | Control | 11 | 42 | 30 | 40 | 0.8 | — | | — |
| HBsAg | CSW | 12 | 30 | 32 | 25 | 0.6 | 5 | C.t. | No |
| | | 13 | 40 | 31 | 34 | 0.7 | 6 | | No |
| | Control | 14 | 26 | | Unknown | | — | | — |

AST, aspartate aminotransferase; ALT, alanine aminotransferase; T-bil, total bilirubin levels; CSW, commercial sex workers; HCV Ab, hepatitis C virus antibody; HBs Ag, hepatitis B surface antigen; Ct, *Chlamydia trachomatis*; Ng, *Neisseria gonorrhoeae*

### DISCUSSION

In Japan, a flourishing sex industry and increasing sexual activities of the young have resulted in considerable infiltration of STD in the general population, especially STD caused by *C. trachomatis* and viruses that are largely asymptomatic. Our previous studies[4,5] using the hybrid capture method demonstrated prevalence of *N. gonorrhoeae* of 4.1%, *C. trachomatis* 13% and HPV 62.1% in CSW in Japan, and a corresponding prevalence of 0.4%, 3% and 8.6% in the general female population. These results show that HPV is the most prevalent STD among Japanese women, especially among CSW. Compared with these infections, our present findings showed no HIV antibody-positive subject and a low prevalence of HBs antigen positivity, and the rate was not particularly high in the CSW group. However, the prevalence of HCV antibody was significantly higher in the CSW group compared to the control group.

In Japan, HIV infection remains relatively low even among the CSW population. Statistics published in July 2000[6] showed a relatively small number of reported cases, with 2367 AIDS patients and 5059 HIV-infected individuals, including 1673 infected by heterosexual contact. However, the existence of a considerable number of undetected cases is suspected. The number of heterosexual transmissions in Japanese males has increased since 1993[2,6]. Once HIV is established in the population, there is a strong possibility that the infection may join the epidemic of other STD, such as *C. trachomatis* infection, and spread rapidly and widely. Although we did not detect HIV antibody-positive individuals in our sample, continuous surveillance is necessary.

The evidence of sexual transmission of HBV was confirmed by a large-scale epidemiological study of male homosexuals in New York[7,8], and hepatitis B infection was established as an STD. In the mid 1970s, a survey of over 600 male homosexuals revealed that 4.6% carried HBs and 51.1% possessed HBs antibody, indicating a history of HBV infection. The most important risk factor was the number of sexual partners[8,9]. In fact, it is difficult to assess accurately how many of the acute hepatitis B cases are sexually transmitted. According to the data of the US Centers for Disease Control, 40–50% of acute hepatitis B cases occurring in the US from 1994–98 were sexually transmitted by heterosexual contact[10].

The prevalence of HBs antigen in the general population in Japan has been reported to be

0.67–2%[11,12]. Although most of the acute hepatitis B cases in adults are considered to be sexually transmitted, unfortunately the actual number and incidence of hepatitis B infections resulting from sexual contact are unknown. A positive HBs antigen reaction but negative antibody reaction is considered to represent either an HBs antigen carrier state or relatively early infection. In the present study, no significant difference in the rate of HBs antigen positivity was observed between the CSW and control subjects. HBs antibody was positive in 23.4% of CSW and 71.8% of control subjects, both showing high rates. The rate in the control group was especially high, probably reflecting a high level of HBV vaccination in hospital staff. Therefore, the high HBV antibody rate in the control group has little implication. A proportion of the CSW were probably also vaccinated against HBV. Therefore, the actual situation of HBV infection becomes even more difficult to assess, and a comparison between the two groups was not possible.

Hepatitis C manifests less severe clinical symptoms than hepatitis B, but the rate of development into a chronic state is markedly high, with high risks of liver cirrhosis and hepatic carcinoma. Elucidation of the transmission route is important, but unfortunately many questions remain unanswered[11,12]. Although blood transfusion is an established route of transmission, infection by this route accounts for only 40% of the total cases in Japan[11,12]. Other possible routes such as perinatal transmission, iatrogenic transmission, hemodialysis-associated transmission, domestic transmission, needle accident, tattooing, needle-sharing among drug addicts, and transmission by sexual contact are being investigated.

In Japan, the prevalence of HCV antibody in the general population is around 1%[11,12]. HCV transmission between husband and wife is not common, but the HCV antibody-positive rate in CSW is eight–ten times higher than in women of the same age group in the general population[11–15]. In our previous results[16], the HCV antibody-positive rate in 203 subjects with high risk for STD was 2.9%. In the present study, the prevalence was 3.2% among CSW, which was significantly higher than that in the control group. Although the positive rates vary among studies due to differences in test method, region and environment, our study demonstrated a significant difference compared to the control group, which agrees with other reports in Japan[14,15,17].

Compared with the data of the United States reported by Gunn and colleagues[18] and those of India reported by Singh and co-workers[19], the prevalence of HIV, HBV and HCV in our present series is low.

CSW in Japan do not necessarily use condoms and have a large number of sexual partners, which make them susceptible to infection by a variety of STD. Individuals with STD such as *C. trachomatis* infection are said to be three–four times more susceptible to HIV infection. Out results also showed that subjects with lesions in the genitals are more susceptible to HBV and HCV infections.

Although the HIV infection rate in the CSW population in Japan remains low, the approval of oral contraceptives in 1999 poses further concern for the spread of STD, and early control measures for AIDS and STD are necessary. STD checkups for CSW alone is inadequate. Health education on the prevention of STD and STD screening for the general public are also required.

### REFERENCES

1. Matsumoto T. Sexually transmitted diseases. *Rinshokensa (Laboratory Investigation)* 1998;42: 1417–27 (in Japanese)
2. Kumamoto E, Tsukamoto Y, Nishitani I, *et al.* Sexually transmitted disease – *Chlamydia* and viruses. *Saishin–Igaku (Modern Medicine)* 1999;54: 196–211 (in Japanese)
3. Nishizono I, Iida S, Suzuki N, *et al.* Rapid and sensitive chemiluminescent enzyme immunoassay for measuring tumor markers. *Clin Chem* 1991;37: 1639–44
4. Ishi K, Suzuki F, Saito A, *et al.* Prevalence of human papillomavirus, *Chlamydia trachomatis* and *Neisseria gonorrhoeae* in commercial sex workers in Japan. *Infect Dis Obstet Gynecol* 2000;26:253–7
5. Ishi K, Suzuki F, Saito A, *et al.* Prevalence of human papillomavirus and its correlation with cervical lesions in commercial sex workers in Japan. *J Obstet Gynecol Res* 2000;4:253–7
6. Japanese Ministry of Health and Welfare. *Report of Trends of AIDS Committee* August 2000
7. Seeff LB, Beebe GW, Hoofnagle JH, *et al.* A serologic follow-up of 1942 epidemic of post-vaccination hepatitis in the United States Army. *N Engl J Med* 1987;316:965–70
8. Hersh T, Melnick JL, Goyal RK, *et al.* Nonparenteral transmission of viral hepatitis B (Australia antigen-associated hepatitis). *N Engl J Med* 1971;285:1363–4
9. Szmuness W, Much MI, Hoofnagle JH, *et al.* On the role of sexual behavior in the spread of hepatitis B infection. *Ann Intern Med* 1975;83:489–95
10. Alter MJ, Ahtone J, Weisfuse I, *et al.* Hepatitis B virus transmission between heterosexuals. *JAMA* 1986;256:1307–10
11. Kashiwagi S. Epidemiology of hepatitis B virus and hepatitis C virus infection. *Jpn J Clin Pathol* 2000; 48:14–9
12. Yoshizawa K. Status of chronic hepatitis in Japan. In *Guidelines of care for chronic hepatitis*. Nihon Kanzobyo Gakkai (Japanese Society of Liver Diseases) 2000, 4–5 (in Japanese)
13. Marusawa H, Uemoto S, Hijikata M, *et al.* Latent hepatitis B virus infection in healthy individuals with antibodies to hepatitis B core antigen. *Hepatology* 2000;31:488–95
14. Kashiwagi S. Status of HIV and STD infections in commercial sex workers. In *1996 Report of HIV Epidemiology Research Group in Health and Welfare Research*. Tokyo, in Japanese 1997
15. Nakashima K. Sexual transmission of hepatitis C virus among female prostitutes and patients with sexually transmitted diseases in Fukuoka, Kyushu, Japan. *Am J Epidemiol* 1992;136:1133–7
16. Kubota T, Iwasa T, Ishi K, *et al.* Transmission of hepatitis C virus in STD high risk group. *Japan Arch Sex Transmit Dis* 1993;4:153–7
17. Kao JH, Chen W, Chen PJ, *et al.* GB virus-C/ hepatitis G virus infection in prostitutes: possible role of sexual transmission. *J Med Virol* 1997;52:381–4
18. Gunn RA, Murray PJ, Ackers ML, *et al.* Screening for chronic hepatitis B and C virus infections in an urban sexually transmitted disease clinic: rationale for integrating services. *Sex Transmit Dis* 2001;28: 166–70
19. Singh S, Thappa DM, Jaisankar TJ, *et al.* Sexual co-transmission of HIV, hepatitis B and hepatitis C viruses. *Sex Transmit Inf* 2000;76:317–8

RECEIVED 05/14/01; ACCEPTED 10/01/01
